# A Machine Learning approach for Total Water storage anomaly eXtension back to 1980 (ML-TWiX)

**DOI:** 10.1038/s41597-026-06604-w

**Published:** 2026-01-29

**Authors:** Peyman Saemian, Mohammad J. Tourian, Karim Douch, James Foster, Junyang Gou, David Wiese, Amir AghaKouchak, Nico Sneeuw

**Affiliations:** 1 Institute of Geodesy, University of Stuttgart, Stuttgart, Germany; 2 European Space Agency, ESRIN, Frascati, Italy; 3 Institute of Geodesy and Photogrammetry, ETH Zurich, Zurich, Switzerland; 4 Jet Propulsion Laboratory, California Institute of Technology, Pasadena, CA USA; 5 Department of Civil and Environmental Engineering, University of California, Irvine, CA USA; 6 Department of Earth System Science, University of California, Irvine, CA USA; 7 United Nations University, Institute for Water, Environment and Health, United Nations University, Ontario, Canada

**Keywords:** Hydrology, Natural hazards

## Abstract

We present ML-TWiX, a global dataset of monthly total water storage anomalies (TWSA) reconstructed from 1980 to 2012, provided on a 0.5^°^ × 0.5^°^ global grid. While the GRACE and GRACE Follow-On satellite missions have provided valuable observations of global TWSA, their combined record spans just over two decades, limiting their utility for long-term climate and hydrological studies. ML-TWiX extends the GRACE-era record into the pre-GRACE period by learning from global hydrological and land surface model simulations using an ensemble of three machine learning models: Random Forest, XGBoost, and Gaussian Process Regression. The three machine learning models were independently used to reconstruct TWSA, and their outputs were subsequently combined through ensemble averaging to produce a unified product with spatially explicit uncertainty estimates. We validated ML-TWiX against multiple independent datasets, including satellite laser ranging, storage deduced from the water mass balance closure, and global mean sea level budget estimates. It provides a continuous reconstruction of global TWSA, enabling a wide range of applications in hydrology, climate science, and water resource assessment.

## Background & Summary

The Gravity Recovery and Climate Experiment (GRACE) satellite and its successor, the GRACE Follow-On (GRACE-FO), have significantly improved our ability to monitor and understand they dynamics of the terrestrial water storage on a global scale^1^. By measuring time-variable gravity, these missions provide direct estimates of total water storage anomalies (TWSA), integrating vertically aggregated changes in groundwater, soil moisture, snow, and surface water. Since their launch, these missions have enabled a wide range of applications in hydrological, climatological, and Earth system science^2–5^. Information derived from GRACE and GRACE-FO (hereafter GRACE(-FO)) has contributed to understanding the freshwater availability^6^, the hydrological impacts of climate extremes, human water use, and processes such as droughts^7^, floods^8^, and sea-level changes^9^.

Despite these achievements, the combined observational period of GRACE and GRACE-FO is limited to two decades of monthly data, with a one-year gap between the missions^10,11^. This relatively short record limits the ability to observe global and regional climate trends in the long term, which is essential for studies on the characterization of drought and long-term patterns of climate change^12^. To compensate for this limitation, several complementary satellite sensors have been employed to estimate TWSA, including Swarm satellites^13–16^, Global Navigation Satellite System (GNSS) inversion^17^, and satellite laser ranging (SLR) satellites^18^. Although space-borne sensors such as Swarm and SLR offer global coverage, their products have lower spatial resolution and accuracy than GRACE, making them insufficient to extend and complement GRACE TWSA fully. Moreover, each spaceborne sensor measures TWSA by relying on different physical principles and observation techniques, which introduce distinct sources of uncertainty and potential bias.

To address the need for an extended TWSA dataset beyond the GRACE mission lifetime, several different methods have been explored, including statistical and machine learning (ML) approaches. Early efforts relied primarily on statistical models. For example, Humphrey *et al*.^19^ developed a statistical reconstruction of the TWSA based on precipitation and temperature data, extending back to 1901, a critical period for assessing long-term variability in global water resources. Building on these efforts, recent studies have investigated ML techniques to better capture the nonlinear and spatiotemporal dynamics of hydrological processes. Some focus on temporal extension, while others target spatial downscaling or gap filling. For example, Sun *et al*.^20^ used a Convolutional Neural Network (CNN) to reconstruct TWSA, utilizing a 3D CNN architecture to learn both temporal and spatial features. Wang *et al*.^21^ applied a Long Short-Term Memory (LSTM) network, achieving high accuracy in modeling temporal dependencies. Jing *et al*.^22^ combined random forest with a spatially moving window structure to capture the spatial heterogeneity of TWSA. These studies show that ML-based reconstructions, including deep learning approaches, generally offer advantages over traditional methods, particularly in flexibility and predictive performance.

Recognizing the strengths of combining diverse modeling strategies, several studies have proposed hybrid frameworks. Sun *et al*.^23^ integrated Deep Neural Network (DNN), Multiple Linear Regression (MLR), and Seasonal AutoRegressive Integrated Moving Average (SARIMAX) to capture both temporal and spatial patterns, resulting in significant improvements over individual methods. Li *et al*.^24^ developed a long-term global reconstruction of TWSA, which is based on machine learning models to reconstruct GRACE-like TWSA with a focus on temporal continuity and regional accuracy. Chandanpurkar *et al*.^25^ developed TWS-CSEOF, which employs cyclostationary empirical orthogonal functions (CSEOFs) and common modes of variability with precipitation and temperature to reconstruct TWSA from 1979 to 2020. GTWS-MLrec, developed by Yin *et al*.^26^, reconstructs TWSA from 1940 to 2022 using machine learning models with various predictors, providing long-term, high-resolution estimates. Recently, Palazzoli *et al*.^27^ introduced GRAiCE, a reconstruction that leverages physical and statistical insights to extend GRACE-like TWSA over a global domain with improved spatial resolution. Most recently, DeepRec was introduced by Gentner *et al*.^28^, which employed a deep learning model that combines CNNs and LSTMs to reconstruct TWSA back to 1941 using climate reanalysis variables and human activity datasets to account for natural variability and anthropogenic effects, respectively.

While these efforts have advanced the state of knowledge, they also face notable limitations. Some methods, such as Humphrey *et al*.^19^, focus primarily on climate-driven variability, which may lead to misrepresentation or omission of long-term trends-particularly those influenced by human activities. Others, like Li *et al*.^24^, extrapolate GRACE-era trends backward without capturing pre-GRACE variability adequately. Many existing datasets exhibit degraded performance in the pre-GRACE era, especially in estimating long-term trends and interannual variability. Furthermore, most studies rely on a single model family or data assimilation scheme without systematically comparing a wide range of methods. To our knowledge, no prior work has explored the combined predictive power of diverse modeling strategies across the statistical-machine learning spectrum, nor has any study integrated the strengths of multiple model families into a unified product. Additionally, validation efforts for the pre-GRACE era have often been limited, and few datasets provide comprehensive or spatially explicit uncertainty estimates.

In this study, we introduce ML-TWiX, a global gridded dataset of total water storage anomalies (TWSA) reconstructed from 1980 to 2012, extending the GRACE record into the pre-GRACE era. Our reconstruction is based on a systematic evaluation of multiple statistical and machine learning methods, ranging from multivariate linear regression (MLR) to advanced nonlinear models. After benchmarking their performance against GRACE observations during the GRACE era, we selected three top-performing methods, namely, Random Forest (RF), Extreme Gradient Boosting (XGB), and Gaussian Process Regression (GPR), and combined them into a single ensemble product. ML-TWiX is provided on a 0.5^°^ global grid and includes uncertainty estimates, enabling users to assess prediction confidence. We validated ML-TWiX against multiple independent references, including satellite laser ranging (SLR), water balance closure (*P* − ET − *R*), and the global mean sea level budget. In all evaluations, ML-TWiX either outperforms or performs comparably to existing long-term reconstructions, offering a reliable and physically consistent dataset for hydrological, climatological, and water resource studies.

## Methods

### Source Data

#### GRACE TWSA

**Fig. 1:**
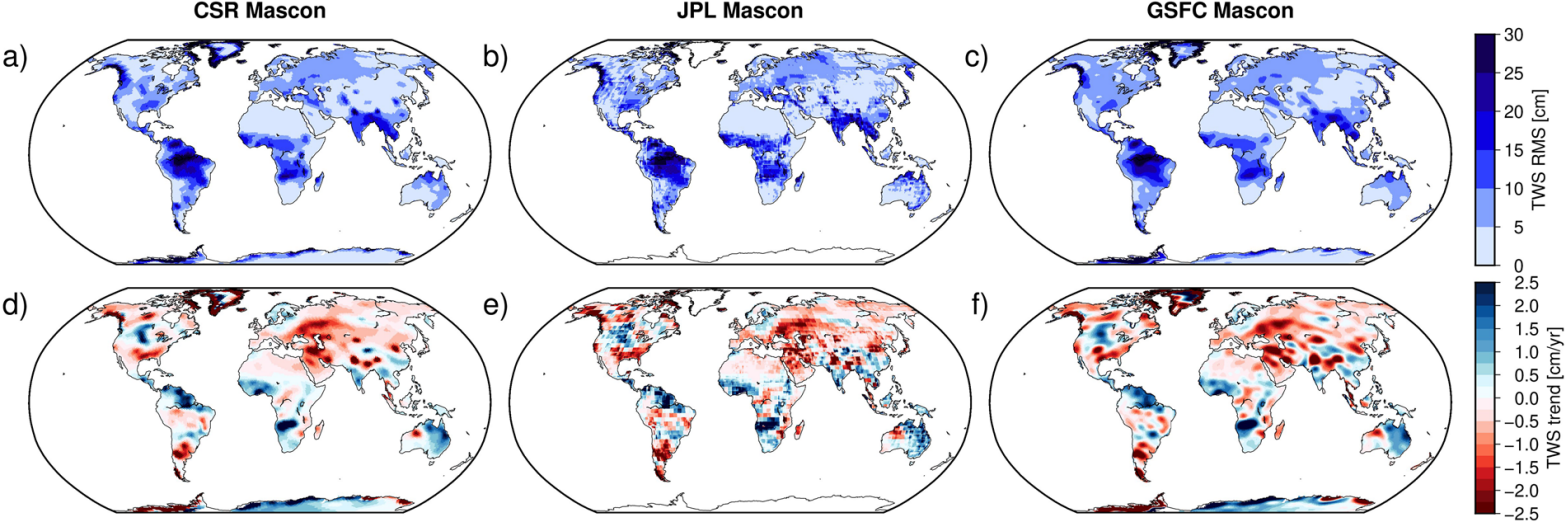
Root Mean Square (RMS) of TWSA (panels **a**-**c**) and linear trend (cm/yr) (panels **d**-**f**) derived from three mascon solutions (CSR, JPL, and GSFC) for the period 2003–2012.

This study uses the latest versions of three GRACE mascon solutions: JPL, CSR, and GSFC. These solutions provide estimates of TWSA with improved spatial accuracy^29^. The data are publicly accessible via their respective repositories: JPL mascon solution via https://grace.jpl.nasa.gov/data/get-data/jpl_global_mascons/, CSR mascon solution via https://www2.csr.utexas.edu/grace/RL06_mascons.html, and GSFC mascon solution via https://earth.gsfc.nasa.gov/geo/data/grace-mascons. While the native spatial resolution of all three products is approximately 300 km  × 300 km, the CSR mascon solution is provided on a denser grid. To ensure consistency across datasets, we resampled the CSR mascon fields onto a common 0.5^°^ grid. This harmonized representation facilitates direct comparison and integration with other datasets used in this study (Table 1) We then computed the ensemble mean of the three solutions. Figure 1 presents the Root Mean Square (RMS) and linear trend of TWSA derived from three mascon solutions (CSR-M, JPL-M, and GSFC-M), illustrating spatial variations in TWSA variability and long-term trends over the study period.

**Table 1:** Mascon solutions used in the study.

Mascon Solution	Center	Gridded Resolution	Reference
JPL Mascon	Jet Propulsion Laboratory (JPL)	0.5^°^ × 0.5^°^	^55–58^
CSR Mascon	Center for Space Research (CSR)	0.25^°^ × 0.25^°^	^59,60^
GSFC Mascon	Goddard Space Flight Center (GSFC)	0.5^°^ × 0.5^°^	^61^

#### Models for TWSA

We employ 13 state-of-the-art data sets of Global Hydrological Models (GHMs), Land Surface Models (LSMs), and atmospheric reanalysis models (see Table 2). Nine multi-decadal global water resources data sets were obtained from the eartH2Observe Water Cycle Integrator (WCI) (http://wci.earth2observe.eulastaccess31May2021). These data sets include PCR-GLOBWB, SURFEX-TRIP, HBV-SIMREG, HTESSEL-CaMa, JULES, LISFLOOD, ORCHIDEE, SWBM, and W3RA. The output of these data sets is available at 0.5^°^ spatial resolution over the period 1980–2012. Additionally, we included the Community Land Model Version 5 (CLM5) with two standard forcing data sets: the Global Soil Wetness Project forcing data set (GSWP3) and CRUNCEP (the combination of the Climate Research Unit (CRU) and the National Centers for Environmental Prediction (NCEP)). The CLM5 data sets are available at 0.5^°^ spatial resolution, covering the period 1901–2014^30^. The CLM5 products are accessible via Earth System Grid (ESG)^31^.

**Table 2:** Summary of global models used in this study.

Model	Category	Time Period	Data Provider	Reference
WGHM	GHM	1980–2012	Goethe University Frankfurt	^62^
PCRGLOB-WB	GHM	1980–2012	Utrecht University (UU)	^63,64^
HBV-SIMREG	GHM	1980–2012	Joint Research Centre (JRC)	^65^
LISFLOOD	GHM	1980–2012	Joint Research Centre (JRC)	^66^
W3RA	GHM	1980–2012	CSIRO^*^	^67^
SWBM	GHM	1980–2012	Simple Water Balance Model	^68,69^
CLM5-CRUNCEP	LSM	1980–2012	ESG^**^ at NCAR	^30^
CLM5-GSWP3	LSM	1980–2012	ESG^**^ at NCAR	^30^
HTESSEL	LSM	1980–2012	ECMWF^***^	^70^
JULES	LSM	1980–2012	Centre for Ecology and Hydrology (CEH)	^71,72^
ORCHIDEE	LSM	1980–2012	French National Centre for Scientific Research	^73^
SURFEX-TRIP	LSM	1980–2012	Meteo France	^74^
ERA5	ReA	1980–2012	ECMWF	^75^

GHM: Global Hydrological Model; LSM: Land Surface Model; ReA: Reanalysis Model. The listed time period corresponds to the common temporal coverage (intersection) across all models used; individual datasets may span a broader time range.

* CSIRO: Commonwealth Scientific and Industrial Research Organisation.

** ESG: Earth System Grid.

*** ECMWF: European Centre for Medium-Range Weather Forecasts.

We also included the latest version of the WaterGAP Global Hydrology Model (WaterGAP v2.2e)^32^, covering the period 1901-2019 at 0.5^°^ spatial resolution. The outputs of WaterGAP v2.2e are available at https://gude.uni-frankfurt.de/entities/researchdata/45867b1e-17f2-4125-a219-ded20bad249e/files. Finally, we used the fifth generation ECMWF atmospheric reanalysis of the global climate (ERA5) at 0.25^°^ spatial resolution, which provides data from 1979 to the present. ERA5 data were downloaded from the Copernicus Climate Change Service (C3S) at ECMWF (https://cds.climate.copernicus.eu; last access: 30 October 2024). For each model, Total Water Storage Anomaly (TWSA) was computed by subtracting the long-term mean over the common period 1980–2012 from the TWS time series. Table 3 highlights the substantial diversity in how the 13 input models represent water-storage components. Only a few models (e.g., CLM5 and PCR-GLOBWB) include a nearly complete set of compartments, whereas others lack key processes such as groundwater, surface water, or human water use. Such structural differences partly explain the spatial variability observed in model performance-for instance, models without snow or SWE layers (like SWBM) tend to perform more poorly in high-latitude basins (see Fig. 5), and models without groundwater or surface-water storage show limitations in heavily regulated or irrigation-dominated regions. Incorporating this heterogeneous ensemble into ML-TWiX allows the machine-learning framework to learn from a broad range of hydrological behaviors, ultimately reducing sensitivity to structural model biases and improving robustness across climate regimes.

**Table 3:** Overview of water storage compartments represented in the 13 models used in this study.

Model	Groundwater	Surface Water	Soil Moisture	Water Use	Reference
WGHM	Yes	Yes	Yes	Yes	^62^
PCR-GLOBWB	Yes	Yes	Yes	Yes	^63,64^
HBV-SIMREG	No	Yes	Yes	No	^65^
LISFLOOD	Yes	Yes	Yes	Yes	^66^
W3RA	No	Yes	Yes	No	^67^
SWBM	No	Yes	Yes	No	^68,69^
CLM5-CRUNCEP	Yes	Yes	Yes	Yes	^30^
CLM5-GSWP3	Yes	Yes	Yes	Yes	^30^
HTESSEL	No	Yes	Yes	No	^70^
JULES	No	Yes	Yes	No	^71,72^
ORCHIDEE	Yes	Yes	Yes	Partial	^73^
SURFEX-TRIP	No	Yes	Yes	No	^74^
ERA5	No	No	Yes	No	^75^

Water use indicates whether anthropogenic withdrawals or irrigation modules are included.

#### SLR-derived TWS

**Fig. 2:**
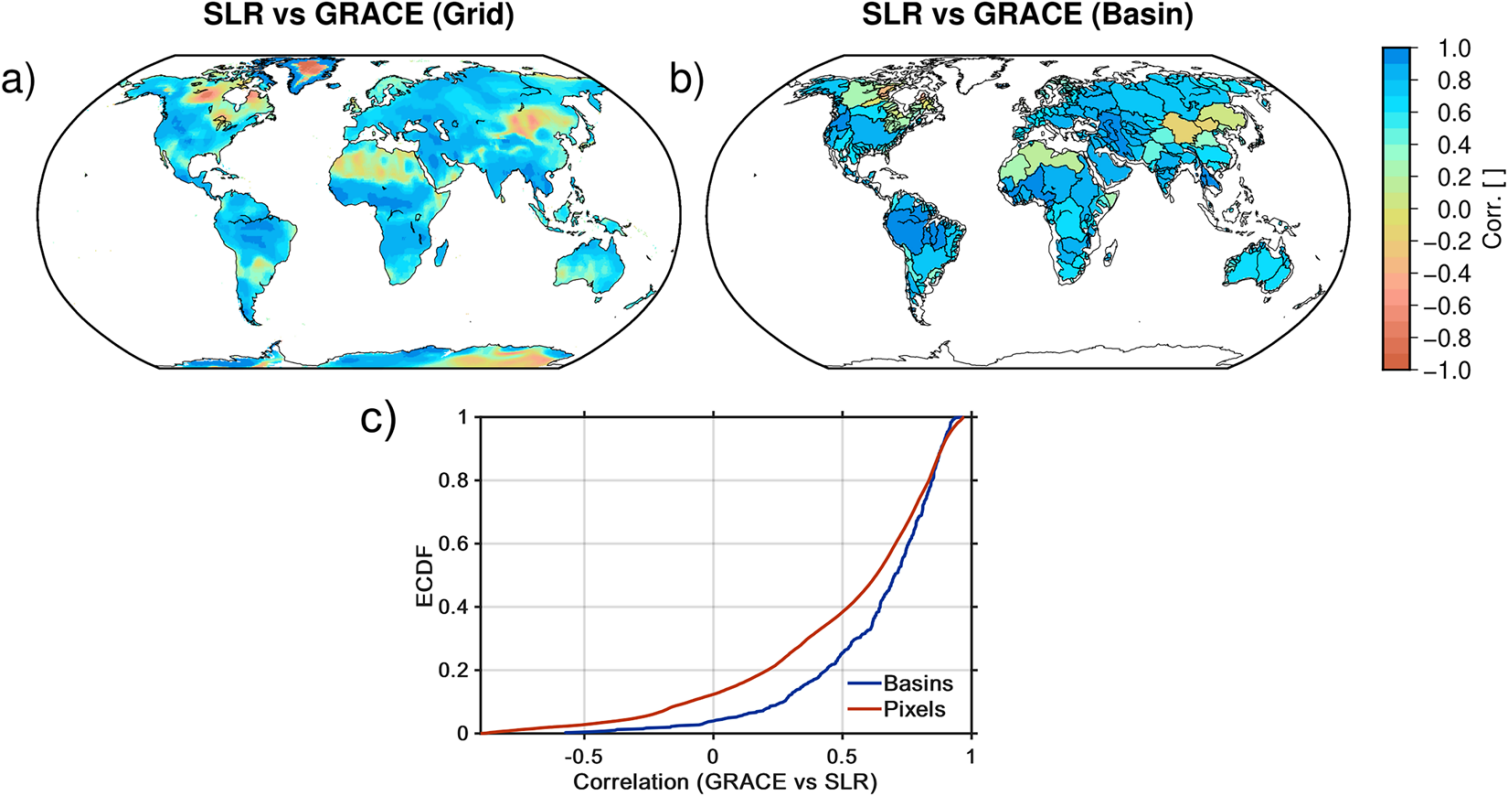
Spatial correlation between GRACE-derived and SLR-based TWSA during the overlapping period (2002–2012): (**a**) grid-scale correlation coefficients computed globally; (**b**) basin-scale correlation coefficients aggregated over major river basins; (**c**) Empirical cumulative distribution functions (ECDFs) of the GRACE-SLR correlation values for both grid cells and basins.

Satellite Laser Ranging (SLR) data provide long-term observations of gravitational field changes, albeit with lower spatial resolution than GRACE. While SLR is most effective for low-degree spherical harmonic coefficients, such as *C*_20_, its sensitivity typically extends to degrees *n* = 5 or *n* = 6, with the potential to partially resolve higher degrees depending on orbital geometry^33^. Recent advances have incorporated auxiliary data and alternative parameterization methods to enhance the spatial resolution of SLR-derived fields. One notable development is the integration of Empirical Orthogonal Functions (EOFs) derived from GRACE into SLR data processing. Using EOFs as base functions, SLR-derived gravity fields can achieve significantly higher resolution, extending to degree *n* = 60. This approach, introduced by Löcher and Kusche^34^, relies on optimizing the SLR solutions using GRACE-derived spatial patterns, improving their capability to estimate TWSA, especially over large basins such as the Amazon. We utilize monthly SLR-based gravity fields covering November 1992 to June 2017. These data are employed to evaluate the reconstructed TWSA during the pre-GRACE era (1992-2002). To obtain TWSA, we removed the mean TWS field over the entire SLR record (1992-2017) from each monthly map. Since the resulting SLR gravity fields inherit the spatial characteristics and noise structure of the GRACE EOFs, we applied an isotropic Gaussian spatial filter with a radius of 300 km. Global TWSA fields are derived at 0.5^°^ global grids, and only the grid cells showing strong agreement (high correlation) with GRACE during the overlapping period are retained for validation in the pre-GRACE era. Figure 2 summarizes the correlation between GRACE-derived and SLR-based TWSA at both grid and basin scales. Panels (a) and (b) show that most land areas exhibit positive correlation during the overlap period, with particularly strong agreement in large basins across North America, Eurasia, and parts of South America. Panel (c) provides the ECDF of these correlations: approximately 30% of basins and 25% of pixels exceed a correlation of 0.8, indicating that SLR captures a substantial portion of the large-scale GRACE signal in many regions. Basin-scale correlations are generally higher than pixel-scale correlations, reflecting the lower noise and stronger spatial coherence of basin-integrated gravitational signals.

#### Water Balance Fluxes

Changes in total water storage can be estimated using the terrestrial water balance equation:

1 $$\frac{{\rm{dTWSA}}}{{\rm{d}}t}=P-{\rm{ET}}-R,$$

where $\frac{{\rm{dTWSA}}}{{\rm{d}}t}$ is the derivative of GRACE-derived TWSA, and *P*, ET, and *R* represent precipitation, evapotranspiration, and runoff, respectively. Given that no single global data set reliably provides high-accuracy water balance fluxes, we derived these fluxes by taking the ensemble mean of selected datasets (Table 4). To achieve this and avoid including lower-quality datasets, we compared each flux dataset with a reference dataset. For precipitation, we used the GPCC version 2022 as a reference. GPCC data are sourced from more than 85,000 stations worldwide, and their use as a reference for comparing precipitation products globally and regionally is well-established^35,36^. For evapotranspiration, we selected the Penman-Monteith-Leuning Evapotranspiration Version 2 (PML-V2) data set, which has demonstrated strong global and regional performance in various studies^37–39^. We incorporated various runoff data sets, including hydrological, reanalysis, and atmospheric models. These data sets were evaluated against the Global RUNoff ENSEMBLE (G-RUN ENSEMBLE), a product developed by Ghiggi *et al*.^40^ using a machine learning approach, 21 different atmospheric forcing datasets, and streamflow observations from the Global Streamflow Indices and Metadata Archive (GSIM).

**Table 4:** Summary of the data sets used in this study to compute the ensemble mean of the water balance fluxes.

	Dataset	Method/Source(s)	Spatial Resolution	Time period	Reference
Precipitation	AgCFSR	G, R	0.25^°^ × 0.25^°^	1980–2010	^76^
	AgMERRA	G, S, R	0.25^°^ × 0.25^°^	1980–2010	^76^
	CHIRPS	G, S, R	0.05^°^ × 0.05^°^	1981–present	^77^
	CRUv4.04	G	0.5^°^ × 0.5^°^	1901–2019	^78^
	GPCCv2020	G	0.25^°^ × 0.25^°^	1982–2019	^79^
	GPCPv2.3	G, S	2.5^°^ × 2.5^°^	1979–present	^80,81^
	GPCP 1DD	G, S	1.0^°^ × 1.0^°^	1996–present	^82^
	GPM IMERG v6 Final	G, S	0.1^°^ × 0.1^°^	2000–present	^83^
	MSWEP v2.8	G, S, R	0.1^°^ × 0.1^°^	1979–2020	^84^
	PERSIANN-CDR	G, S	0.25^°^ × 0.25^°^	1983–present	^85^
	PREC/L	G	0.5^°^ × 0.5^°^	1948–present	^86^
	TRMM-3B42-adj	G, S	0.25^°^ × 0.25^°^	1998–2019	^87^
	UDELv5.01	G	0.5^°^ × 0.5^°^	1900–2017	^88^
ET	ERA5	R	31 km	1979–present	^75^
	FluxCom	R	31 km	1979–present	^89^
	P-LSH	R	31 km	1979–present	^90^
	PML-v2	R	31 km	1979–present	^39^
Runoff	G-RUN	R	31 km	1979–present	^40^
	SURFEX-TRIP	R	31 km	1979–present	^65^
	W3RA	R	31 km	1979–present	^67^

The abbreviations indicate the primary data source for each dataset: G (gauge-based), S (satellite-based), and R (reanalysis).

Each data set for each flux was evaluated against its respective reference using three commonly applied metrics: correlation coefficient (CC), root mean square error (RMSE), and Kling-Gupta efficiency (KGE). Data sets with KGE > 0.5 (KGE > 0.3 for runoff) and RMSE < 10 % of the mean annual value were selected. The final estimation for each flux was obtained as the ensemble mean of the selected datasets in each grid cell. All selected data sets maintain a spatial resolution of 0.5^°^, and daily values were aggregated by summing all days within each calendar month to obtain monthly flux totals. When required, data sets were interpolated to 0.5^°^ resolution using a nearest-neighbor approach, which does not affect the ranking of data sets^41^.

**Fig. 3:**
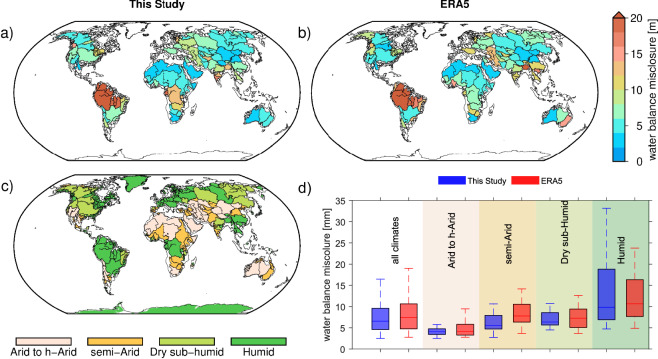
Assessment of water balance misclosure and hydroclimatic context across major global river basins (excluding Greenland and Antarctica): (**a**) spatial distribution of water balance misclosure and fluxes (precipitation, evapotranspiration, and runoff) estimated in this study; (**b**) corresponding misclosure using fluxes from ERA5 reanalysis; (**c**) Köppen-Geiger climate classification map highlighting major climate zones; and (**d**) bar plots comparing average misclosure from panels (**a**) and (**b**) across different climate categories. This comparison illustrates the improvement in water balance closure achieved using the ensemble-based flux estimates under varying climatic conditions.

To evaluate the effectiveness of the derived fluxes in closing the terrestrial water balance, we compared the residual misclosure-defined as the difference between $\frac{{\rm{dTWSA}}}{{\rm{d}}t}$ and *P* − ET − *R*-using two approaches: the ensemble-based fluxes developed in this study, and the corresponding fluxes directly from ERA5. The comparison is summarized in Fig. 3, which shows boxplots of the absolute misclosure error across all grid cells and for different climatic regions. Across all regions, the median misclosure using our ensemble-based fluxes is significantly lower (6.6 mm/month) compared to that from ERA5 fluxes (7.5 mm/month). This improvement is more pronounced in regions with higher water variability. For example, in Category 1 regions-those with reliable GRACE-SLR agreement-the median misclosure drops from 4.2 mm/month (ERA5) to 4.1 mm/month using our approach. Similarly, in Category 2 regions, the misclosure improves from 5.6 mm/month (ERA5) to 4.7 mm/month. These results indicate that our carefully selected and combined flux datasets more accurately capture the monthly variations in total water storage, thereby reducing uncertainty in global water balance assessments.

#### Global Sea Level Change

To evaluate ML-TWiX at the global scale, we employed a sea level budget approach that links TWS changes to global mean sea level (GMSL) variations. Changes in land water storage impact sea level through mass exchange between the continents and the oceans. We used the sea level budget reconstruction provided by Frederikse *et al*.^42^, which integrates multiple observational datasets and model estimates into a probabilistic framework, offering component-wise contributions to sea level change from 1900 to 2018. We isolated the TWS component by subtracting the contributions of thermosteric expansion (*Δh*_thermosteric_) and mass changes in the Greenland (*Δh*_GrIS_) and Antarctic (*Δh*_AIS_) ice sheets from the total GMSL (*Δh*_GMSL_). This residual captures the combined effect of TWS and land glaciers, as glacier contributions are implicitly included in GRACE-based observations and thus in our reconstruction. Formally, this derived TWS estimate is expressed as:

2 $$\Delta {h}_{{\rm{TWS,}}{\rm{derived}}}=\Delta {h}_{{\rm{GMSL}}}-\Delta {h}_{{\rm{GrIS}}}-\Delta {h}_{{\rm{AIS}}}-\Delta {h}_{{\rm{thermosteric}}}$$

To enable a consistent comparison with ML-TWiX, we applied a sign inversion to the derived TWS estimates, accounting for the inverse relationship between land water storage and ocean mass. The same procedure was applied to the other reconstructed datasets to enable a consistent comparison with ML-TWiX in closing the global sea level budget.

For the post-1992 period, we additionally incorporated the GMSL time series from satellite altimetry, which provides improved accuracy compared to tide-gauge reconstructions. Specifically, we used the Version 5.2 dataset provided by NASA GSFC^43^, which integrates altimeter measurements from TOPEX/Poseidon, Jason-1, OSTM/Jason-2, Jason-3, and Sentinel-6 into a unified record corrected for inter-mission biases and geophysical effects. This makes it particularly suitable for evaluating global-scale hydrological contributions to sea level during the satellite era.

#### GRACE-Like TWS Reconstruction Products

We compared our results with the following datasets of reconstructed GRACE-like TWSA products:

- GRACE-Rec^19,44^: This data set uses a statistical model trained with GRACE observations to reconstruct past TWS changes. The latest version (version 3) of the GRACE-Rec dataset can be downloaded from 10.6084/m9.figshare.7670849.
- Li *et al*.^24^: This dataset relies on machine learning models to reconstruct GRACE-like TWSA, focusing on temporal continuity and regional accuracy, particularly in significant hydrological changes. The dataset can be accessed via 10.5061/dryad.z612jm6bt.
- Chandanpurkar *et al*.^25^: This data set uses empirical cyclostationary orthogonal functions (CSEOF) and common modes of variability with precipitation and temperature to reconstruct the TWSA from 1979-2020. The resultant TWS reconstruction from this study is available for download at https://zenodo.org/records/6659543.
- GTWS-MLrec^26^: This data set uses machine learning models with various predictors to reconstruct TWSA from 1940 to 2022, providing long-term, high-resolution estimates. The data set is available at 10.5281/zenodo.10040927.
- GRA*i*CE^45^: This is a collection of four global monthly TWSA reconstructions spanning from 1984 to 2021, at a spatial resolution of 0.5^°^. These reconstructions were developed using Long Short-Term Memory (LSTM) and Bidirectional LSTM (BiLSTM) neural networks. GRA*i*CE is available for download on Zenodo at 10.5281/zenodo.10953658.
- Mandal *et al*.^46^: This data set provides a global reconstruction of monthly TWSA from 1960 to 2022 at a spatial resolution of 0.5^°^. The reconstruction, termed BNML_TWSA, uses optimal predictors selected via a Bayesian network from land surface model outputs (e.g., GLDAS), meteorological variables, and climate indices such as the Oceanic Niño Index and Dipole Mode Index. A suite of machine learning models, including CNN, SVR, ETR, and stacking ensemble regression, was tested at each grid cell, with Extra Trees Regression (ETR) emerging as the most effective for most regions. The dataset is available at 10.6084/m9.figshare.25376695.
- DeepRec^28^): This dataset provides a global reconstruction of monthly TWSA from 1941 to 2023 at 0.5^°^ spatial resolution. DeepRec combines climate reanalysis and human activity data as inputs into a spatiotemporal deep learning model that integrates convolutional neural networks (CNNs) and long short-term memory (LSTM) networks. The model is trained on GRACE(-FO) data and produces both TWSA and associated uncertainty estimates using a deep ensemble framework. The reconstruction is evaluated against GRACE, satellite laser ranging, the global sea level budget, and the terrestrial water balance. DeepRec is available on Zenodo via 10.5281/zenodo.15681366.

#### ML-TWiX reconstruction framework

**Fig. 4:**
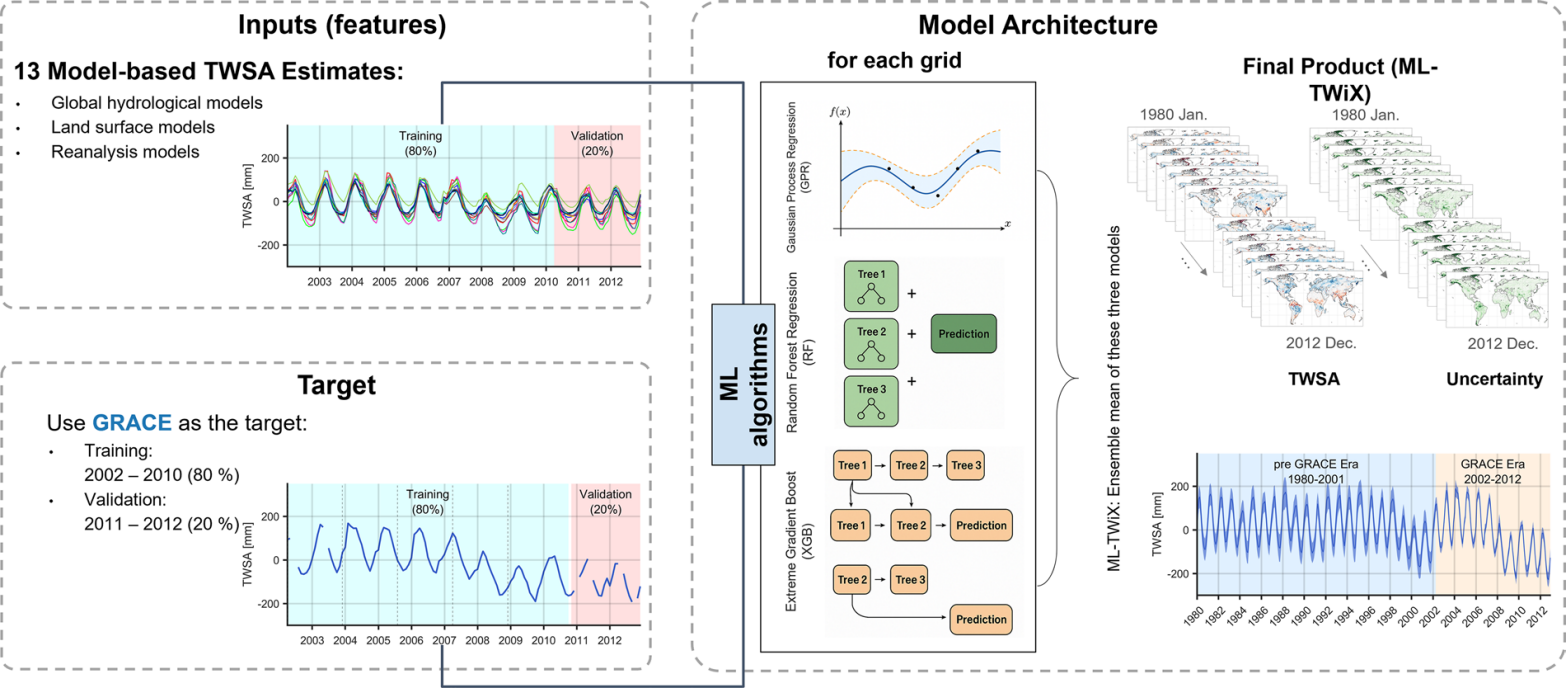
Flowchart of the ML-TWiX reconstruction framework. Thirteen model-based TWSA estimates from global hydrological models, land surface models, and reanalysis datasets serve as inputs. GRACE observations (2002–2012) are used as training and validation targets. Three machine learning algorithms- RF, XGB, and GPR-which demonstrated the highest performance against GRACE during the satellite period, are used to model TWSA at each grid cell. The final ML-TWiX product is generated by taking the ensemble mean of the predictions from these three models. The dataset is provided at 0.5^°^ spatial resolution and includes corresponding uncertainty fields.

The ML-TWiX reconstruction framework is summarized in Fig. 4. It integrates thirteen global model-based TWSA time series as predictors and uses GRACE observations from April 2002 to December 2012 as the training and validation target. We reconstructed monthly TWSA at 0.5^°^ resolution over global land areas (excluding Greenland and Antarctica) for the pre-GRACE era (January 1980 - March 2002). The reconstruction was carried out using a suite of statistical and machine learning techniques, all trained on GRACE-era data and applied to model-based inputs to hindcast TWSA for the earlier period. After evaluating multiple methods, three machine learning models-Random Forest (RF), eXtreme Gradient Boosting (XGB), and Gaussian Process Regression (GPR)-were selected for their superior agreement with GRACE. These models were applied independently at each grid cell, and their outputs were ensemble-averaged to generate the final reconstruction. The reconstruction period (1980–2012) is determined by the temporal overlap of the thirteen model-based inputs, which provide continuous and internally consistent global coverage beginning in 1980. The GRACE era (2002–2012) is used exclusively for training and validation to ensure that ML-TWiX functions strictly as a hindcasting framework rather than replacing GRACE or GRACE-FO during their operational periods. Extending the reconstruction further back in time (e.g., to the 1940s) would require re-running all contributing models with harmonized forcing fields-an effort beyond the scope of the current study but a promising direction for future versions of the dataset. In addition to the reconstructed TWSA, ML-TWiX provides spatially explicit uncertainty estimates. In the following sections, we briefly describe each of the statistical and machine learning methods used in the reconstruction:

#### (1) Statistical Methods

These methods rely on linear combinations of model estimates, either with equal or optimized weights. Let **X**(*t*) = [*X*_1_(*t*), *X*_2_(*t*), …, *X*_*n*_(*t*)] denote the set of *n* model-based TWSA estimates used as inputs at time *t*, with *n* = 13 in our case. The Ensemble Mean (EM) served as a benchmark method, representing the naive average of all model estimates:

3 $${\widehat{Y}}_{{\rm{EM}}}(t)=\frac{1}{n}\mathop{\sum }\limits_{i=1}^{n}{X}_{i}(t)$$

The Weighted Least Squares (WLS) method computes an optimal linear combination of models based on predefined weights. From now on we call the result of WLS, Ensemble Weighted Mean (EWM). The weights were derived from the inverse of the absolute root mean square error (RMSE) between each model and GRACE over the training period:

4 $${w}_{i}=\frac{1}{| {\rm{RMSE}}({X}_{i},Y)| },\quad {\widehat{Y}}_{{\rm{WLS}}}(t)=\frac{{\sum }_{i=1}^{n}{w}_{i}{X}_{i}(t)}{{\sum }_{i=1}^{n}{w}_{i}}$$

While using the inverse of RMSE squared would theoretically align with inverse-variance weighting, we chose 1/RMSE to avoid over-amplifying small performance differences, which can lead to over-weighting a single model. This more moderate weighting strategy provides greater stability and preserves model diversity in the ensemble, helping to mitigate overfitting and maintain robustness across regions and time periods.

Moreover, we employed the Multivariate Linear Regression (MLR). To this end, we fit a multivariate linear model by minimizing the squared error between GRACE and the weighted sum of models:

5 $${\widehat{Y}}_{{\rm{MLR}}}(t)={\beta }_{0}+\mathop{\sum }\limits_{i=1}^{n}{\beta }_{i}{X}_{i}(t)$$

where *β*_0_ is a single intercept shared across all predictors. The coefficients {*β*_*i*_} were estimated using ordinary least squares on the training period.

To ensure physical interpretability and avoid negative contributions, we also fit a constrained version of the MLR, enforcing non-negativity (Non-Negative Least Squares: NNLS) on the coefficients by adding an inequality constraint:

6 $${\widehat{Y}}_{{\rm{NNLS}}}(t)={\beta }_{0}+\mathop{\sum }\limits_{i=1}^{n}{\beta }_{i}{X}_{i}(t),\quad \,{\rm{subject\; to}}\,{\beta }_{i}\ge 0\,\forall i$$

#### (2) Machine Learning Methods

To account for potential nonlinear interactions among the thirteen model-based TWSA estimates, in addition to statistical methods, we employed several supervised learning algorithms capable of modeling nonlinear relationships between the model-based estimates and GRACE observations. All machine learning models were implemented in MATLAB, and their corresponding hyperparameters were tuned using Bayesian optimization with *k*-fold cross-validation (with *k* = 5). Each model was trained to approximate the mapping:

7 $${f}_{\theta }:{{\mathbb{R}}}^{n}\to {\mathbb{R}},\quad {\widehat{Y}}_{{\rm{ML}}}(t)={f}_{\theta }({X}_{1}(t),{X}_{2}(t),\ldots ,{X}_{n}(t))$$

where *f*_*θ*_ is the learned regression function with parameters *θ*, mapping the input model-based estimates **X**(*t*) to the target GRACE TWSA.

##### Decision Tree Regression (DT)

Decision Tree Regression uses a recursive binary partitioning of the input space. In our case, the input at each time step is a 13-dimensional vector representing the TWSA estimates from the thirteen model-based sources. Each internal node of the tree performs a binary split based on a threshold applied to one of the input values. The tree continues splitting until a stopping criterion is met, resulting in *M* terminal nodes (leaves). Each leaf corresponds to a distinct region *R*_*m*_, and is assigned a constant prediction:

8 $${\widehat{Y}}^{{\rm{DT}}}(t)=\mathop{\sum }\limits_{m=1}^{M}{c}_{m}\cdot {\mathbb{I}}({\bf{X}}(t)\in {R}_{m}),$$

where *R*_*m*_ is the *m*-th region (leaf), *c*_*m*_ is the predicted value for that region, and ${\mathbb{I}}(\,\cdot \,)$ is the indicator function. DT is interpretable, captures nonlinear relationships, and serves as a building block for ensemble methods such as Random Forest Regression (RF) and Extreme Gradient Boosting (XGB).

##### Support Vector Regression (SVR)

Support Vector Regression (SVR) is a kernel-based supervised learning method derived from Support Vector Machines. It estimates a regression function by minimizing a loss that penalizes deviations larger than a specified margin *ϵ*, while also controlling model complexity via regularization. SVR seeks a function that fits the training data within an *ϵ*-insensitive tube while maximizing generalization.

Given input ${\bf{X}}(t)\in {{\mathbb{R}}}^{13}$, SVR predicts TWSA using the form:

9 $${\widehat{Y}}^{{\rm{SVR}}}(t)=\mathop{\sum }\limits_{i=1}^{N}{\alpha }_{i}\,k({{\bf{X}}}_{i},{\bf{X}}(t))+b,$$

where *α*_*i*_ are support vector coefficients, **X**_*i*_ are the support vectors from the training set, *b* is the bias term, and *k*( ⋅ , ⋅ ) is a kernel function. In this study, we used the radial basis function (RBF) kernel:

10 $$k({\bf{x}},{{\bf{x}}}^{{\prime} })=\exp \left(-\frac{\parallel {\bf{x}}-{{\bf{x}}}^{{\prime} }{\parallel }^{2}}{2{\sigma }^{2}}\right)$$

Hyperparameters such as the kernel bandwidth *σ*, the regularization parameter *C*, and the margin *ϵ* were tuned using Bayesian optimization. SVR is effective in modeling nonlinear relationships and provides good generalization, particularly for small- to medium-sized datasets.

##### Random Forest Regression (RF)

Random Forest is an ensemble learning method that builds multiple regression trees using bootstrapped subsets of the training data and aggregates their predictions to reduce variance and improve generalization. For each tree, a random sample (with replacement) is drawn from the original training set. At each node split, a randomly selected subset of the input variables (i.e., the 13 model-based TWSA estimates) is considered as candidates for splitting. This process introduces diversity among the trees and helps decorrelate their predictions. The final prediction is obtained by averaging the outputs of all trees in the ensemble:

11 $${\widehat{Y}}^{{\rm{RF}}}(t)=\frac{1}{B}\mathop{\sum }\limits_{b=1}^{B}{T}_{b}({\bf{X}}(t)),$$

where *T*_*b*_(⋅) is the *b*-th regression tree, and *B* is the total number of trees. RF is robust to overfitting, can model complex nonlinear relationships, and is relatively insensitive to the scaling of input data.

##### Extreme Gradient Boosting (XGB)

XGB is a tree-based ensemble method that builds regression trees sequentially, where each new tree is trained to reduce the errors (residuals) of the previous ensemble. The model is trained by minimizing a regularized objective function, which balances data fitting and model complexity:

12 $${\mathcal{L}}=\sum _{t}\ell (Y(t),\widehat{Y}(t))+\sum _{k}\Omega ({f}_{k}),$$

where ${\mathcal{L}}$ is the total loss, *ℓ* is the data loss (we used squared error in this study), and Ω(*f*_*k*_) is a regularization term that penalizes the complexity of each tree *f*_*k*_ (e.g., number of leaves and their weights). The prediction is computed as the sum of outputs from all trees:

13 $${\widehat{Y}}^{{\rm{XGB}}}(t)=\mathop{\sum }\limits_{k=1}^{K}{f}_{k}({\bf{X}}(t)),\quad {f}_{k}\in {\mathcal{F}},$$

where ${\mathcal{F}}$ denotes the space of regression trees. In our implementation, we used the default squared-error loss and optimized hyperparameters such as tree depth, learning rate, and regularization strength using Bayesian optimization. XGB is known for its high predictive performance, and the built-in regularization terms help reduce overfitting, especially when working with high-dimensional or noisy data.

##### Gaussian Process Regression (GPR)

GPR is a Bayesian non-parametric regression model that defines a distribution over functions. It assumes the output values follow a joint multivariate Gaussian distribution with a kernel function *k*(⋅ , ⋅) capturing similarity between inputs:

14 $$Y(t) \sim {\mathcal{G}}{\mathcal{P}}(m({\bf{X}}(t)),\,k({\bf{X}}(t),{\bf{X}}({t}^{{\prime} })))$$

where *m*(⋅) is the mean function which is often set to zero, assuming the data have been centered, or when the focus is on modeling the covariance structure rather than the absolute signal level. *k*(⋅ , ⋅) is a covariance kernel, such as the squared exponential kernel (also called radial basis function):

15 $$k({\bf{x}},{{\bf{x}}}^{{\prime} })={\sigma }_{f}^{2}\exp \left(-\frac{1}{2{\ell }^{2}}\parallel {\bf{x}}-{{\bf{x}}}^{{\prime} }{\parallel }^{2}\right)$$

where **x** and ${{\bf{x}}}^{{\prime} }$ are two input vectors of model-based TWSA values (i.e., 13-dimensional vectors corresponding to different time steps), ${\sigma }_{f}^{2}$ is the signal variance, and *ℓ* is the length-scale parameter controlling smoothness. In our implementation, we used Gaussian Process Regression with Bayesian hyperparameter optimization to select the optimal kernel and its parameters. The candidate kernels included the squared exponential, Matérn class with smoothness parameters $\nu =\frac{5}{2}$ and $\nu =\frac{3}{2}$, and the rational quadratic kernel. Hyperparameters such as the noise level (*σ*_*n*_), kernel scale (length-scale *ℓ*), and kernel function were optimized using 5-fold cross-validation. The predictive distribution at a new input X^*^ is Gaussian with mean and variance:

16 $${\widehat{Y}}^{{\rm{GPR}}}({{\bf{X}}}^{* })={\mu }_{* }={{\bf{k}}}_{* }^{\top }{(K+{\sigma }_{n}^{2}I)}^{-1}{\bf{Y}}$$

17 $${\rm{Var}}[{\widehat{Y}}^{{\rm{GPR}}}({{\bf{X}}}^{* })]=k({{\bf{X}}}^{* },{{\bf{X}}}^{* })-{{\bf{k}}}_{* }^{\top }{(K+{\sigma }_{n}^{2}I)}^{-1}{{\bf{k}}}_{* }$$

where *K* is the covariance matrix of the training data, and k_*_ is the vector of covariances between X^*^ and the training inputs, **Y** is the vector of GRACE-derived TWSA values at the corresponding training time steps. GPR provides both predictions and uncertainty estimates, is flexible with a principled probabilistic foundation, and performs well on small- to medium-sized datasets.

#### Estimating uncertainty

Quantifying the uncertainty of reconstructed TWSA is crucial for informing downstream users about the confidence and reliability of the estimates, especially when analyzing long-term hydrological changes under non-stationary climate and anthropogenic conditions. In ML-TWiX, we adopted an ensemble-based approach to characterize uncertainty, drawing inspiration from deep ensemble frameworks^47–49^, but implemented with diverse machine learning regressors in a modular and interpretable way.

We applied each selected machine learning model to generate *M* = 5 independent realizations per 0.5^°^ grid cell by varying initialization settings while keeping the training data fixed. Each realization yields a TWSA estimate ${\widehat{\mu }}_{m}$ for each time step and location, from which the model-specific is calculated from:

18 $${\widehat{\mu }}_{k}(t)=\frac{1}{M}\mathop{\sum }\limits_{m=1}^{M}{\widehat{\mu }}_{k,m}(t).$$

While GPR analytically provides a predictive variance for each estimate, we deliberately applied the same ensemble-based resampling approach to GPR as to the other models. This decision ensures methodological consistency and facilitates a fair comparison of uncertainty magnitudes across models. Additionally, bootstrapping captures variability arising from different initializations, which complements the epistemic uncertainty already embedded in GPR.

Following a mixture-based ensemble approach described in Lakshminarayanan *et al*.^47^, we aggregated the predictions from the three selected machine learning models by averaging their outputs. The ensemble mean is:

19 $${\widehat{\mu }}^{* }(t)=\frac{1}{K}\mathop{\sum }\limits_{k=1}^{K}{\widehat{\mu }}_{k}(t).$$

This ensemble mean serves as the final reconstructed TWSA, combining the strengths of each model. The total uncertainty σ^*^ implicitly contains both uncertainty components: (1) *Aleatoric uncertaintyσ*_Ale_, capturing the intrinsic variability or noise within each model, derived from the average of model-specific variances:

20 $${\sigma }_{{\rm{Ale}}}^{2}(t)=\frac{1}{K}\mathop{\sum }\limits_{k=1}^{K}\left(\frac{1}{M}\mathop{\sum }\limits_{m=1}^{M}{\widehat{\sigma }}_{k,m}^{2}(t)\right),$$

and, (2) *Epistemic uncertaintyσ*_Epi_, representing the disagreement between model predictions, calculated as the variance of the model means:

21 $${\sigma }_{{\rm{Epi}}}^{2}(t)=\frac{1}{K}\mathop{\sum }\limits_{k=1}^{K}{\left({\widehat{\mu }}_{k}(t)-{\widehat{\mu }}^{* }(t)\right)}^{2}.$$

The final predictive uncertainty reported in ML-TWiX is:

22 $${\sigma }^{* }(t)=\sqrt{{\sigma }_{{\rm{Ale}}}^{2}(t)+{\sigma }_{{\rm{Epi}}}^{2}(t)}.$$

This ensemble-based uncertainty quantification offers two key advantages: (i) it allows spatially and temporally resolved uncertainty estimates, and (ii) it improves robustness when the characteristics of the input data differ between the training and application periods, as each model realization is independently optimized. While this approach assumes that the spread among predictions captures both types of uncertainty-a simplification compared to fully probabilistic deep learning models-it offers a practical and interpretable framework consistent with prior work^50,51^.

## Data Records

The ML-TWiX data set^52^ is available via the DaRUS repository at 10.18419/DARUS-5233. It provides monthly global total water storage anomalies (TWSA) from January 1980 to December 2012 on a 0.5^°^ regular latitude-longitude grid, excluding Greenland and Antarctica. The data set is stored in NetCDF-4 format and adheres to CF (Climate and Forecast) metadata conventions to ensure compatibility with standard analysis tools.

The main data file contains the following variables:

- TWSA: Reconstructed total water storage anomaly in millimeters,
- uncertainty: Combined uncertainty associated with each TWSA estimate.
- lat, lon, time: Coordinate variables.

All variables are provided at a monthly resolution and stored in a single NetCDF file named ML-TWiX_1980_2012.nc. A README file accompanies the data set, documenting the file structure, variable definitions, units, and spatial/temporal coverage. To facilitate dataset usage, example scripts in MATLAB and Python are provided for loading, visualizing, and extracting time series from the NetCDF file.

## Technical Validation

### Data Set Results

The results are presented in four main parts. First, we evaluate the performance of the individual input models by comparing them to GRACE TWSA observations at the basin scale, providing a baseline for reconstruction. Second, we assess the reconstruction methods described in the Methods section-including both statistical and machine learning approaches-based on their ability to reproduce GRACE TWSA across major global river basins. This evaluation helps determine their suitability for a large-scale application. Third, based on this assessment, we select three well-performing machine learning methods and apply them to reconstruct TWSA at a 0.5^°^ grid resolution over global land areas (excluding Greenland and Antarctica). Finally, we quantify the uncertainty of the ML-TWiX reconstruction by analyzing the spatial and temporal variability of prediction uncertainty across scales. For basin-level analysis, we use shapefiles from the Global Runoff Data Center (GRDC) representing major river basins worldwide.

#### (I) Modeled TWSA vs GRACE-dervied TWSA (2002–2012)

**Fig. 5:**
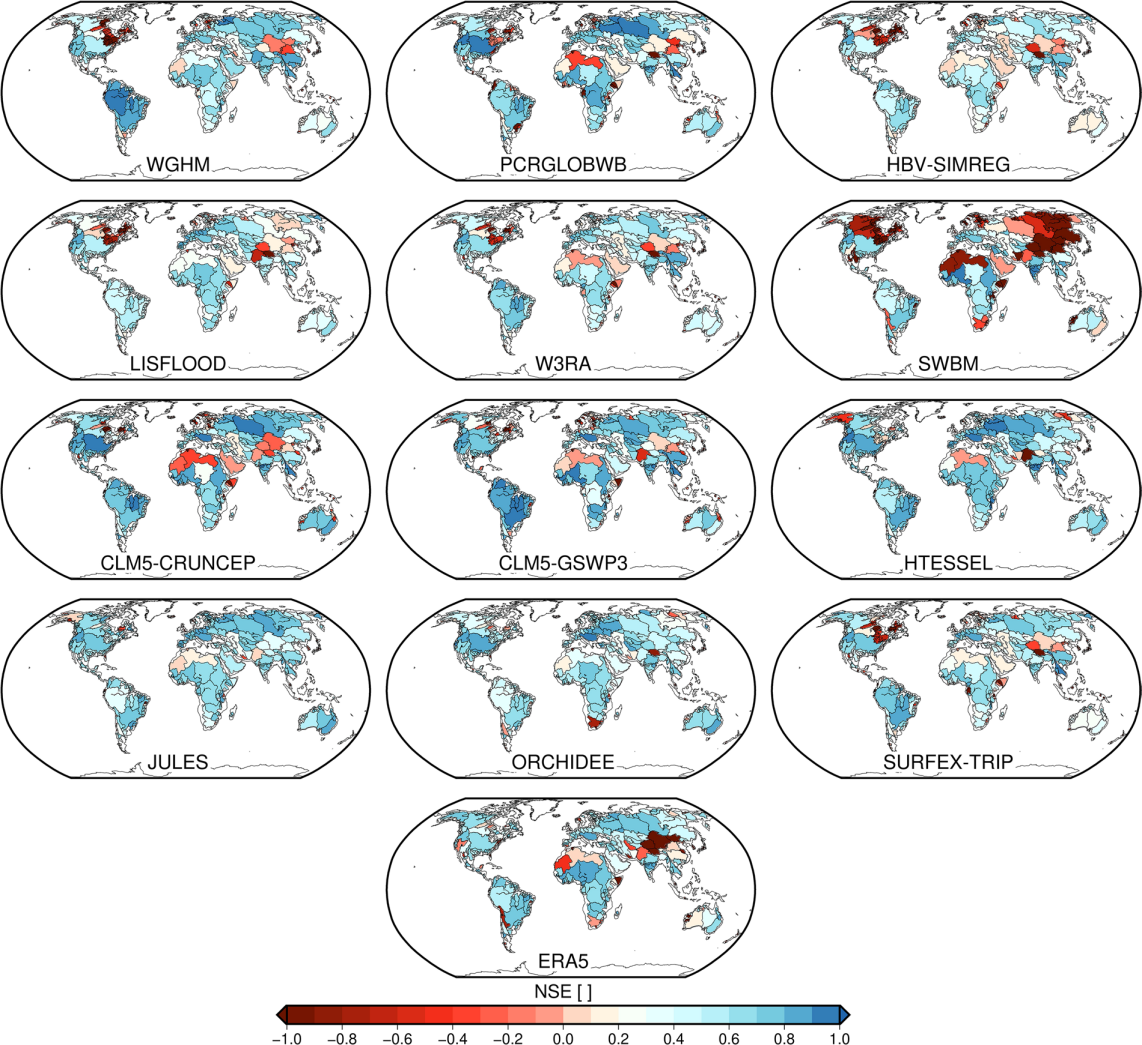
Basin-scale Nash-Sutcliffe Efficiency (NSE) values for various models compared against GRACE TWSA across major global river basins (excluding Greenland and Antarctica). NSE values range from  − *∞* to 1, with 1 indicating perfect agreement, 0 meaning the model performs no better than the observed mean, and negative values indicating poor performance.

We evaluated the performance of each model in estimating TWSA by comparing it with GRACE-derived TWSA across major river basins, using the Nash-Sutcliffe Efficiency (NSE) as the primary metric (Fig. 5). NSE values range from  −*∞* to 1, where 1 indicates perfect agreement, 0 implies the model performs no better than the mean of the observations, and negative values suggest poor model performance. The NSE ranges exhibited significant variability across basins and climate (see Fig. 3(c)for the distribution of the basins’ climate classes), reflecting differences in the models’ ability to represent hydrological dynamics. Some models performed consistently well in specific climates, while others showed more variable skills. For tropical-dominated basins, models such as SWBM, WaterGAP, and HBV-SIMREG frequently achieved higher NSE values, indicating their skill in capturing water storage variations. In contrast, CLM5-CRUNCEP and ERA5 showed moderate performance, with noticeable underperformance in some basins. The low NSE values observed in some basins can be explained with prior studies showing that several global hydrological models tend to underestimate long-term trends and show seasonal amplitude and phase offsets relative to GRACE across different climate zones^53,54^.

Arid basins posed challenges for most models, generally yielding lower NSE values. However, W3RA and PCR-GLOBWB demonstrated relatively better alignment with GRACE TWSA, likely due to their ability to represent water fluxes in sparsely vegetated regions. In temperate basins, LISFLOOD, HTESSEL, and ORCHIDEE emerged as strong performers, consistently achieving high NSE values. This suggests that these models effectively capture the seasonal and interannual variability characteristic of temperate climates. For cold-climate basins, where snow and ice dynamics play a critical role, JULES, CLM5-GSWP3, and SURFEX-TRIP exhibited higher NSE values, highlighting their ability to incorporate snow-related processes. Other models, such as HBV-SIMREG, showed mixed results, indicating potential limitations in representing cryospheric influences.

The results highlight that no single model consistently outperforms others across all basins and climate types. While WaterGAP and HBV-SIMREG perform well in specific regions, ERA5 and CLM5-CRUNCEP exhibit considerable variability depending on the basin and climate. These results demonstrate that each model is best suited to particular hydroclimatic conditions and struggles to generalize across diverse environments. This underscores the need for a multi-model integration approach, which we explore in the next section.

#### (II) ML-TWiX TWSA vs GRACE-derived TWSA

**Fig. 6:**
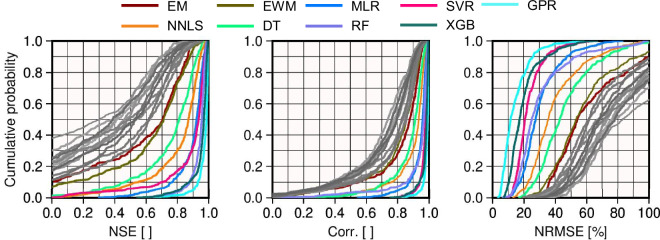
Cumulative Distribution Functions (CDFs) of three performance metrics-Nash-Sutcliffe Efficiency (NSE), Correlation (Corr), and Normalized Root Mean Square Error (NRMSE)-comparing reconstructed TWSA methods with GRACE-derived TWSA from April 2002 to December 2012. Gray lines represent the TWSA time series from the 13 individual input models (GHMs, LSMs, and reanalysis products) that serve as predictors in the reconstruction methods.

The performance of the eight different methods, along with the simple ensemble mean (the naive method) for combining models to reconstruct TWSA, was evaluated using three metrics: correlation coefficients, NRMSE, and NSE. The NRMSE was computed as the RMSE between the reconstructed and GRACE TWSA time series, normalized by the standard deviation of the GRACE TWSA over the evaluation period. The cumulative distribution functions (CDFs) of these metrics are demonstrated for 403 basins over the period 2002–2012 to compare the approaches (see Fig. 6). The results reveal significant improvements in reconstruction accuracy when using advanced approaches over the Ensemble Mean and each of the individual models across all metrics. Among advanced methods, GPR consistently leads in all three metrics, achieving the highest correlation with GRACE (mean of 0.99), the lowest normalized RMSE (mean of 7 %), and the highest NSE (mean of 0.98). These results underscore GPR’s ability to balance accuracy and reliability in both linear and non-linear settings, making it the most robust approach for reconstructing TWSA. XGB closely follows, with slightly higher errors and marginally lower correlation and NSE values. RF also performs well but trails behind GPR and XGB, particularly in reducing NRMSE. In contrast, linear approaches such as MLR and NNLS, while showing improvements over the Ensemble Mean, fail to match the performance of non-linear machine learning methods. MLR achieves a high correlation (mean of 0.94) and NSE (mean of 0.89), but its relatively higher NRMSE (mean of 0.40) suggests limitations in capturing non-linear variability in TWSA dynamics. NNLS, though conceptually appealing for ensuring positive contributions, exhibits even lower correlations and higher errors relative to other methods. These results collectively indicate that while linear methods may work reasonably well in regions with simpler hydrological processes, advanced non-linear methods such as GPR and XGB are better suited for capturing the complexity of TWSA variations.

#### (III) Results for the selected methods

**Fig. 7:**
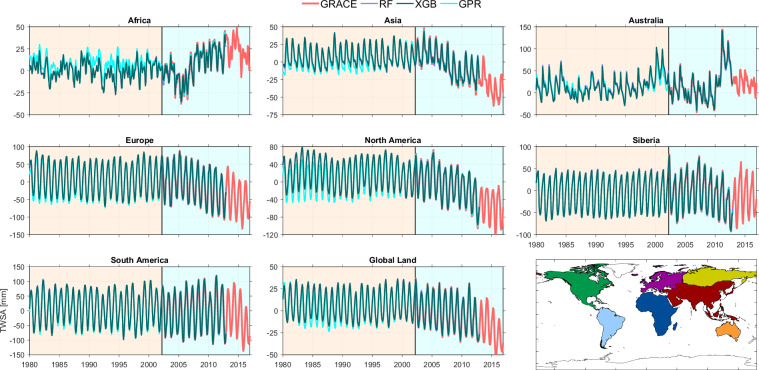
Time series of GRACE TWSA alongside three reconstructed TWSA methods—RF, XGB, and GPR-plotted over individual continents and global land.

**Fig. 8:**
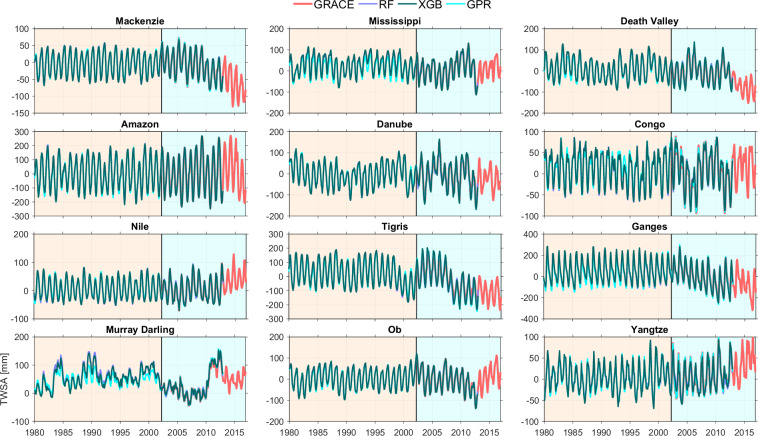
Time series of GRACE TWSA alongside three reconstructed TWSA datasets-RF, XGB, and GPR-plotted over selected major river basins.

Our global reconstruction of TWSA spanning 1980 to 2012, cover all global land areas except Greenland and Antarctica. Here we present the reconstructed TWSA of each selected methods at three spatial scales: sub-continental, basin, and grid levels. We focus on the three best-performing methods-RF, XGB, and GPR-identified based on their agreement with GRACE during the training period (Fig. 6). Figure 7 shows the time series of GRACE and reconstructed TWSA aggregated over continents and global land, while Fig. 8 provides basin-scale time series for selected river basins. In both cases, the reconstructions effectively reproduce the seasonal cycles and long-term trends observed in the GRACE record.

#### (IV) Estimated uncertainty

**Fig. 9:**
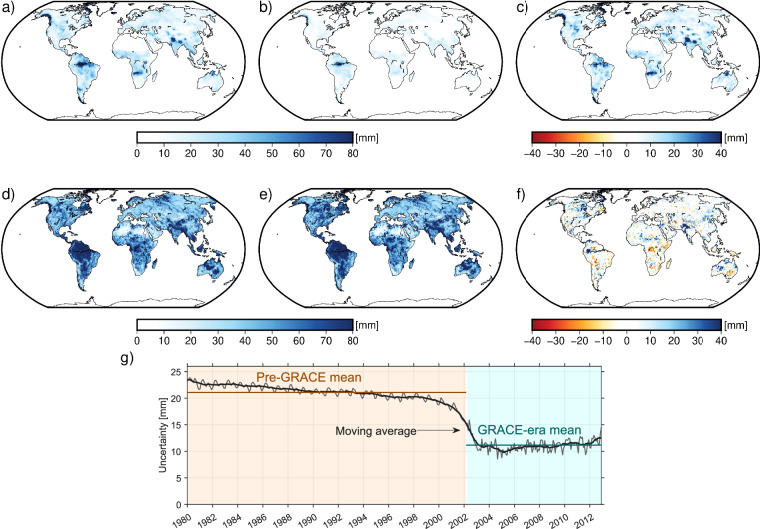
Uncertainty estimates from the ML-TWiX reconstruction. Panel (**a**) shows the spatial distribution of mean predictive uncertainty during the pre-GRACE era (January 1980 to March 2002), and panel (**b**) shows the same for the GRACE era (April 2002 to December 2012). Panel (**c**) shows the spatial distribution of mean predictive uncertainty during the GRACE era, subtracted from mean predictive uncertainty during the pre-GRACE era. Panels (**d**) and (**e**) show the spatial distribution of the mean standard deviation among the 13 input models during the pre-GRACE and GRACE eras, respectively, while panel (**f**) presents their difference. Panel (**d**) (**g**) presents the time series of mean uncertainty over global land areas (excluding Greenland and Antarctica), along with a moving average. The two periods are highlighted in different colors, and the mean uncertainty for each period is indicated.

Figure 9 shows the spatial distribution of mean uncertainty in the ML-TWiX dataset for the pre-GRACE (January 1980-March 2002; panel a) and GRACE (April 2002-December 2012; panel b) periods. Uncertainty is highest in regions like Amazon Basin, Central Africa, and parts of Southeast Asia- areas typically characterized by large hydrological variability-for example, monsoon-dominated basins, snow-influenced high-latitude regions, and strong wet-dry seasonal regimes-which also exhibit greater divergence among the input models in both the amplitude and timing of TWSA variations. In contrast, arid and semi-arid regions generally exhibit lower uncertainty due to their more stable water storage dynamics. While the overall spatial patterns are similar across both periods, the GRACE era shows noticeably lower uncertainty magnitudes. The reduction reflects improved agreement among input models during the GRACE era. It also results from the fact that this period was used for model training, leading to inherently smaller discrepancies between model predictions. Panel (d) of Fig. 9 shows the time series of mean uncertainty over global land (excluding Greenland and Antarctica) from 1980 to 2012. The time series shows a clear reduction in uncertainty starting around 2002, coinciding with the onset of the GRACE era. The mean uncertainty during the pre-GRACE period (1980–2002) is approximately 21 mm, while it decreases to about 11 mm during the GRACE era (2002–2012). The higher mean uncertainty in the pre-GRACE period reflects the extrapolation of models beyond the training window, where no direct GRACE observations constrain the estimates, leading to greater disagreement among models. During the GRACE era, training on observed data reduces epistemic uncertainty by ~65%, resulting in smaller model spread and higher confidence in the estimates. Panels (d)-(e) further demonstrate that the spread among the 13 input models is substantially larger than the final predictive uncertainty in ML-TWiX, indicating that the machine-learning ensemble effectively reduces structural disagreement inherited from the models. Panel (f) shows the spatial pattern of this reduction, highlighting regions where model disagreement decreased during the GRACE era as well as areas where it remained elevated or even increased. Moreover, across all validation results, we include the ensemble mean to enable direct comparison, showing that ML-TWiX consistently exhibits reduced spread relative to both individual inputs and the simple model mean.

### Data Set Validation

We assess the quality of the reconstructed TWSA from the ML-TWiX ensemble using independent datasets and complementary observations. To facilitate a broader intercomparison, we also include other reconstructed TWSA products. The validation is organized into four components: (I) comparison with GRACE-derived TWSA during the overlap period between GRACE and ML-TWiX (April 2002-December 2012), to assess consistency with the reference observations; (II) comparison with TWSA inferred from satellite laser ranging (SLR)-based sea level budget residuals; (III) evaluation of the ability of reconstructed TWSA to close the terrestrial water balance (via comparison with residual fluxes *P* − ET − *R*); and (IV) assessment of the reconstructed TWSA’s role in closing the global sea level budget.

#### (I) Comparison of ML-TWiX TWSA with GRACE-derived TWSA

**Fig. 10:**
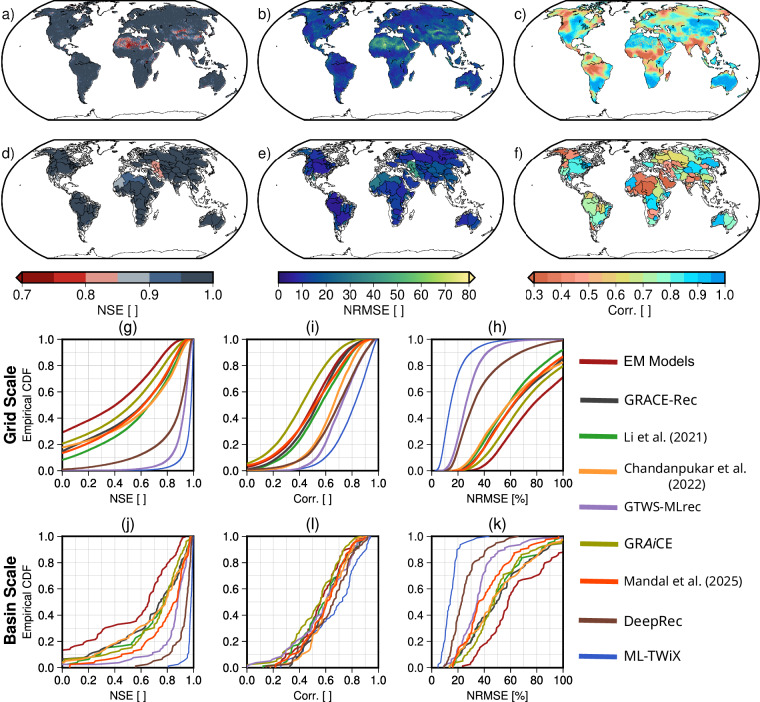
Validation of the ML-TWiX TWSA against GRACE TWSA using three performance metrics: NSE, NRMSE, and correlation (calculated on residuals, i.e., detrended and deseasonalized). Panels (**a**)–(**c**) show grid-scale results for NSE, NRMSE, and correlation, respectively. Panels (**d**)–(**f**) show the same metrics at the basin scale for 79 large basins (>200,000 km^2^). Panels (**g**)–(**i**) present empirical CDFs of ML-TWiX and other reconstructed datasets for NSE, NRMSE, and correlation at the grid scale; panels (**j**)–(**l**) show the corresponding results at the basin scale.

Figure 10 presents a comprehensive assessment of the ML-TWiX dataset in comparison to GRACE TWSA observations using three statistical metrics: correlation coefficient, NRMSE, and NSE. Note that the correlation coefficient is applied to the residual signal after removing the linear trend, annual, and semiannual components. The first row shows the grid-scale global maps of these metrics for ML-TWiX, highlighting strong agreement with GRACE across most regions, with particularly high correlations (>0.9) and NSE values over temperate and tropical zones. The second row aggregates these results at the basin scale, limited to 79 river basins larger than 200,000 km^2^ as GRACE-based TWSA estimates are known to be more reliable over basins of this size or larger. ML-TWiX maintains high performance in these regions. Many basins in the Amazon, Congo, and Ganges exhibit correlation values above 0.95 and NSE values exceeding 0.9, underscoring the dataset’s ability to represent spatial and temporal water storage dynamics.

The third and fourth rows display the empirical CDFs of the three validation metrics across all grid cells (third row) and major river basins (fourth row), comparing ML-TWiX with seven existing reconstruction datasets, including GRACE-Rec, GRAiCE, GRACE-MLrec, DeepRec, and others. ML-TWiX consistently ranks among the top performers. At the basin scale, ML-TWiX achieves a mean NSE of 0.96, a mean NRMSE of 13.6%, and a mean correlation of 0.99, clearly outperforming other datasets (e.g., DeepRec with NSE = 0.91, NRMSE = 21.9%, Corr = 0.97; GRACE-Rec with NSE = 0.67, NRMSE = 51.9%, Corr = 0.88). Notably, ML-TWiX outperforms all others in terms of NSE and NRMSE, indicating better accuracy in capturing basin-level residual variability.

Further analysis based on basin climate classification (from the Köppen-Geiger system) reveals systematic patterns in model performance (see Fig. 3(c) for the distribution of the basins’ climate classes). ML-TWiX demonstrates particularly high skill in humid tropical (H) and temperate climates, with multiple basins showing NSE  > 0.95 and correlation  > 0.98. In contrast, performance declines in arid (Dry sH) and cold climates, though ML-TWiX still surpasses most other datasets in these regions. This highlights the robustness of the ML-TWiX reconstruction across diverse hydroclimatic settings, while also acknowledging persistent challenges in modeling TWSA in sparsely gauged or snow-dominated environments.

#### II Comparison of ML-TWiX TWSA with SLR-derived TWSA

**Fig. 11:**
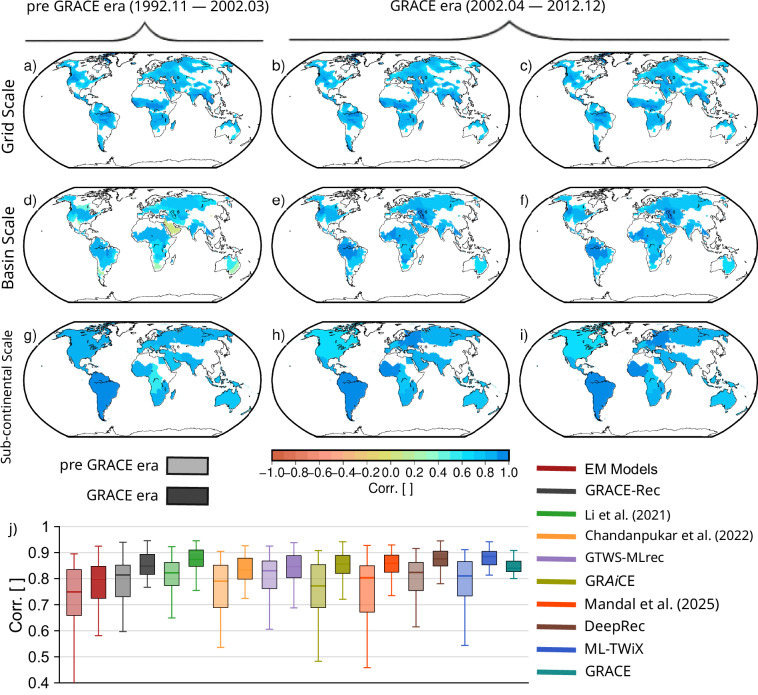
Spatial correlation between reconstructed TWSA and satellite laser ranging (SLR)-derived TWSA. Panels (**a**)–(**c**), (**d**)–(**f**), and (**g**)–(**i**) show correlations at grid, basin, and sub-continental scales, respectively. The first two columns represent ML-TWiX for the pre-GRACE era (Nov. 1992-Mar. 2002; **a, d, g**) and GRACE era (Apr. 2002-Dec. 2012; **b, e, h**). The third column (**c, f, i**) shows GRACE-SLR correlations during the GRACE era. Panel (**j**) presents boxplots of basin-scale correlations between SLR and each reconstruction (including ML-TWiX, GRACE, and the ensemble mean of models) for both periods.

To assess the consistency of ML-TWiX, we compared the globally integrated TWSA from ML-TWiX with TWSA inferred from SLR, both during the GRACE era (April 2002-December 2012) and the pre-GRACE era (November 1992-March 2002) (Fig. 11). The correlation analysis was performed at three spatial scales-grid, basin, and sub-continental-using only regions where the correlation between GRACE and SLR exceeded 0.8. This threshold helps identify locations where the SLR signal is robust enough for evaluation (see Fig. 2 for the full correlation distribution). During the GRACE era, ML-TWiX showed strong agreement with the SLR-derived TWSA across all scales, with correlation values (median  = 0.88) closely matching those between GRACE and SLR itself (median = 0.88), thereby validating the consistency of the reconstruction.

The lower panels of the figure summarize the correlation results at the basin scale through boxplots, shown separately for the GRACE and pre-GRACE eras. As before, only basins with GRACE-SLR correlation greater than 0.8 are included. ML-TWiX exhibits the highest median correlation in both periods (0.88 and 0.82 for GRACE and pre-GRACE eras, respectively), highlighting its ability to generalize beyond the training window. DeepRec performs comparably during the GRACE era (0.88) and maintains relatively strong skill before GRACE (0.82) as well. In contrast, GRACE-Rec and GRAiCE demonstrate strong agreement during the GRACE era (0.85 and 0.87, respectively) but exhibit substantial degradation in performance in the pre-GRACE period (0.77 and 0.85), suggesting greater reliance on temporal continuity with GRACE era trends. Other datasets, such as Li *et al*. (2021) and GTWS-MLrec, show moderate-to-limited performance across both periods.

#### III Performance in closing the water balance

**Fig. 12:**
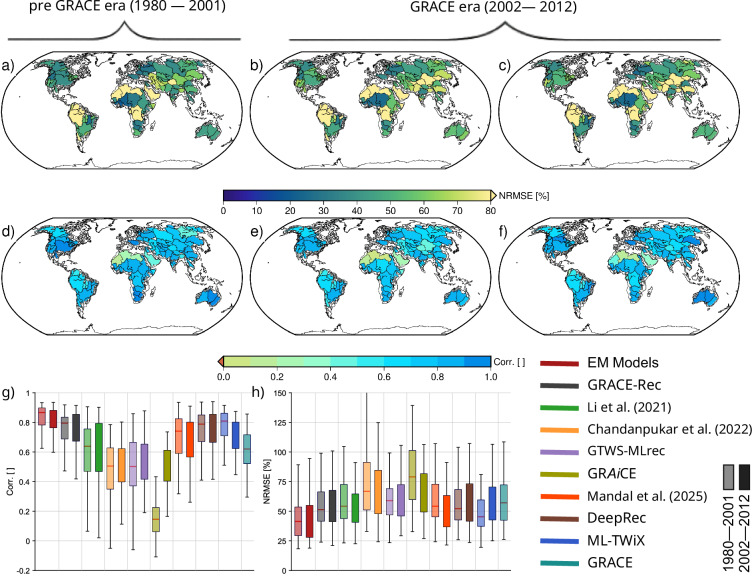
Validation of reconstructed TWSA derivatives against water balance residuals (*P* − ET − *R*) over basins bigger than 200,000 km^2^. Panels (**a**)–(**c**) show NRMSE [%] and (**d**)–(**f**) show correlation between derivative of TWSA and *P* − ET − *R* for ML-TWiX during 1980–2001 (**a, d**), ML-TWiX during 2002–2012 (**b, e**), and GRACE during 2002–2012 (**c, f**). Panels (**g**) and (**h**) show boxplots of basin-level correlation and NRMSE, respectively, for all datasets during the two time periods.

To assess how well reconstructed TWSA data sets capture the water cycle dynamics, we evaluated the correlation and normalized RMSE (NRMSE, in %) between the temporal derivative of TWSA and the residual fluxes (*P* − *ET* − *R*) across major river basins (>200,000 km^2^), over two distinct periods: the GRACE era (2002-2012) and the pre-GRACE era (1980-2001) (Fig. 12). This dual-period framework not only benchmarks model accuracy but also assesses how consistently each reconstruction performs across decades with varying observational coverage.

The first two rows of Fig. 12 present spatial maps of NRMSE and correlation for ML-TWiX and GRACE. The first column shows ML-TWiX performance in the pre-GRACE era, the second column its performance in the GRACE era, and the third column presents GRACE itself during the GRACE era. These maps highlight that ML-TWiX maintains high correlations and low errors across many large basins, particularly in humid and dry sub-humid regions, even in the absence of GRACE data. ML-TWiX demonstrates comparable performance to GRACE in many regions during 2002–2012 and exhibits consistent spatial patterns extending back to 1980, underscoring its robustness across time.

The third row (panels g and h) presents boxplots summarizing basin-level statistics. During the GRACE era, the ensemble mean (EM) of model-based TWSA shows the best performance overall, with a median correlation of 0.96 and an NRMSE of 39%. Among reconstructed datasets, DeepRec ranks highest (Corr = 0.93, NRMSE = 54%), followed closely by ML-TWiX (Corr = 0.91, NRMSE = 54%) and GRACE-Rec (Corr = 0.91, NRMSE = 49%). In the pre-GRACE era, ML-TWiX achieves the highest correlation (Corr = 0.94), outperforming all other reconstructions, including DeepRec (Corr = 0.92). Its NRMSE remains low (45%), highlighting ML-TWiX’s strong generalizability and stability beyond the training period.

This consistency is particularly noteworthy given the decline in data quality further back in time. For reconstruction datasets that extend further back in time (e.g., DeepRec, GRACE-CSEOF), satellite-based observations were sparse or nonexistent before 1979, and many gauge-based datasets had limited spatial coverage or quality. These constraints likely explain the broader spread in performance across datasets in the earlier decades. Notably, datasets like GRACE-CSEOF exhibit substantial degradation in the pre-GRACE period, with correlation dropping by more than 0.08 and NRMSE increasing by nearly 18 percentage points.

Spatial patterns support these findings: humid and dry sub-humid basins tend to show higher agreement, while arid and hyper-arid basins display larger errors and more variability, reflecting the challenges of capturing fluxes in regions where both precipitation and evapotranspiration inputs are highly uncertain. Overall, both DeepRec and ML-TWiX demonstrate strong and consistent performance across time periods, with ML-TWiX particularly standing out in the pre-GRACE era for its ability to maintain accuracy despite the lack of satellite-era training data.

#### IV Performance in Closing the Sea Level Budget

**Fig. 13:**
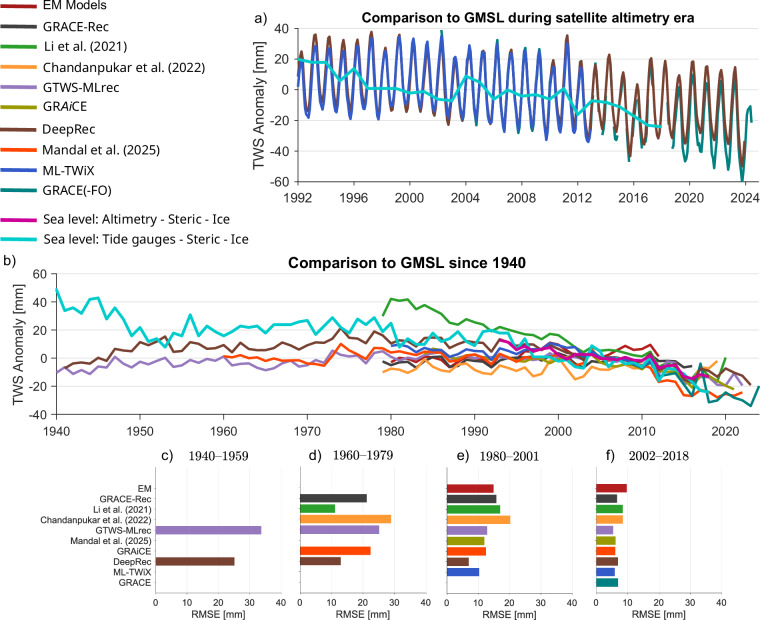
Evaluation of reconstructed global TWSA against the global sea level budget. Panel (**a**) shows the comparison of annual TWS anomalies from ML-TWiX and sea level-derived estimates after 1992, alongside monthly records from GRACE, ML-TWiX, and DeepRec. Panel (**b**) extends this comparison to annual TWS from multiple reconstructions and the sea level estimate starting in 1941. Panels (**c**)–(**f**) present the RMSE between global mean TWSA from each dataset and the residual sea level estimate (i.e., sea level minus steric height and polar ice contributions), shown in four roughly 20-year segments, with panel (**f**) covering the GRACE(-FO) period.

To assess the ability of ML-TWiX and other reconstructions to represent long-term variations in TWSA, we compared globally averaged TWSA time series against an independent estimate derived from global mean sea level (GMSL), after removing the contributions of thermosteric expansion and ice mass loss from Greenland and Antarctica (Fig. 13). This comparison includes two GMSL sources: satellite altimetry for the post-1992 period and tide gauge records for earlier decades. ML-TWiX shows strong agreement with both GMSL-derived estimates across the full evaluation period. During the GRACE era (2002-2012), when altimetry-based estimation is also available, ML-TWiX achieves the lowest misfit among all evaluated datasets, followed closely by DeepRec. This suggests that ML-TWiX effectively captures global-scale TWS variability where GRACE data and high-quality remote sensing inputs are available.

Between 1980 and 2001, DeepRec slightly outperforms ML-TWiX in closing the sea level budget, with both datasets showing noticeably lower error than other reconstructions. Further back in time, only DeepRec and GTWS-MLrec provide continuous reconstructions before 1960. However, the performance of all data sets, including these two, gradually declines as the time horizon extends into earlier decades. This degradation reflects increased uncertainty in the underlying input data: before 1979, remote sensing contributions were limited or absent, and available in situ gauge observations-while valuable-may not have been sufficiently dense or consistent to support high-resolution reconstruction of key water balance components. Overall, while ML-TWiX and DeepRec both demonstrate strong skill from the 1980s onward, their relative performance varies across periods. The comparison highlights the inherent challenges in extending TWSA reconstructions into the pre-satellite era and underscores the importance of accounting for observational uncertainty when interpreting long-term trends. It is important to mention that the current temporal extent of ML-TWiX is limited by the availability of input model simulations rather than by the reconstruction methodology itself. As longer records from hydrological and land surface models become available, ML-TWiX can be readily extended further back in time using the same framework. We note that any reconstruction method trained primarily on the GRACE era implicitly assumes that the statistical relationships learned from the GRACE period remain valid outside it. This assumption may be violated if long-term changes in climate, land-surface processes, or anthropogenic water management introduce nonstationarity in TWS dynamics. Such nonstationarity can challenge the extrapolation skill of machine-learning reconstructions. By incorporating independent validation sources (SLR, water-balance residuals, sea level budget) and using multiple hydrological and LSM inputs, we partially assess and mitigate these risks, although they cannot be fully removed.

## Data Availability

The ML-TWiX dataset is publicly available via the DaRUS repository at the University of Stuttgart 10.18419/DARUS-5233. The dataset includes monthly global total water storage anomalies (TWSA) from January 1980 to December 2012 on a 0.5^°^ grid, together with associated uncertainty estimates. The repository contains two NetCDF files: ML_TWiX_ensemble_v1_0.nc (TWSA) and ML_TWiX_ensemble_uncertainty_v1_0.nc (uncertainty), as well as example MATLAB and Python scripts (read_NetCDFs_MATLAB.m, read_NetCDFs_Python.py) for reading and visualizing the data.

## Code availability

All data processing and reconstruction methods were implemented using MATLAB R2022. The scripts used to read and visualize the data set are publicly available alongside the ML-TWiX dataset in the DaRUS repository: 10.18419/DARUS-5233.
